# Host-Specific *Bacteroides* Markers-Based Microbial Source Tracking in Aquaculture Areas

**DOI:** 10.1264/jsme2.ME17166

**Published:** 2018-06-01

**Authors:** Hye Young Ko, Kyuseon Cho, SungJun Park, Jin Hwi Kim, Joo-Hyon Kang, Yong Seok Jeong, Jong Duck Choi, Yongsik Sin, Cheonghoon Lee, GwangPyo Ko

**Affiliations:** 1 Department of Environmental Health Sciences, Graduate School of Public Health, Seoul National University 1 Gwanak-ro, Gwanak-gu, Seoul Republic of Korea; 2 N-Bio, Seoul National University 1 Gwanak-ro, Gwanak-gu, Seoul Republic of Korea; 3 Department of Civil and Environmental Engineering, Dongguk University 1 Pildong-ro, Jung-gu, Seoul Republic of Korea; 4 Department of Biology, College of Sciences, Kyung Hee University 26 Kyungheedae-ro, Dongdaemun-gu, Seoul Republic of Korea; 5 Department of Seafood Science and Technology, Gyeongsang National University 38 Cheondaegukchi-gil, Tongyeong-si, Gyeongsangnam-do Republic of Korea; 6 Department of Environmental Engineering & Biotechnology, Mokpo National Maritime University 91, Haeyangdaehak-ro, Mokpo-si, Jeollanam-do Republic of Korea; 7 Institute of Health and Environment, Graduate School of Public Health, Seoul National University 1 Gwanak-ro, Gwanak-gu, Seoul Republic of Korea; 8 Center for Human and Environmental Microbiome, Seoul National University 1 Gwanak-ro, Gwanak-gu, Seoul Republic of Korea

**Keywords:** fecal contamination, geographic information system, host-specific *Bacteroides* markers, microbial source tracking, noroviruses

## Abstract

Various waterborne pathogens originate from human or animal feces and may cause severe gastroenteric outbreaks. *Bacteroides* spp. that exhibit strong host- or group-specificities are promising markers for identifying fecal sources and their origins. In the present study, 240 water samples were collected from two major aquaculture areas in Republic of Korea over a period of approximately 1 year, and the concentrations and occurrences of four host-specific *Bacteroides* markers (human, poultry, pig, and ruminant) were evaluated in the study areas. Host-specific *Bacteroides* markers were detected widely in the study areas, among which the poultry-specific *Bacteroides* marker was detected at the highest concentration (1.0–1.2 log_10_ copies L^−1^). During the sampling period, high concentrations of host-specific *Bacteroides* markers were detected between September and December 2015. The host-specific *Bacteroides* marker-combined geospatial map revealed the up-to-downstream gradient of fecal contamination, as well as the effects of land-use patterns on host-specific *Bacteroides* marker concentrations. In contrast to traditional bacterial indicators, the human-specific *Bacteroides* marker correlated with human specific pathogens, such as noroviruses (*r*=0.337; *P*<0.001). The present results indicate that host-specific *Bacteroides* genetic markers with an advanced geospatial analysis are useful for tracking fecal sources and associated pathogens in aquaculture areas.

Feces represent a major source of water contamination because it contains various microbial pathogens. Most waterborne human pathogens originate from feces, including noroviruses and food-poisoning bacteria, and may spread widely and cause severe gastroenteric diseases (5, 21, 36). Waterborne disease cases have been increasingly reported in Republic of Korea since 2009, and 422 cases of waterborne disease outbreaks, accounting for 8% of all infectious disease cases, occurred in Republic of Korea in 2015 (Gyeongnam development institute. 2011. A study on improvement of water quality in Milyanggang. Gyeongsangnam-do, Republic of Korea, in Korean) (Korea centers for disease control and prevention. 2015. Infectious disease surveillance yearbook. Chungcheonbuk-do, Republic of Korea, in Korean). The southern part of the Korean peninsula is a major aquaculture area, producing more than 70% of domestic seafood for Republic of Korea (Ministry of Ocean and Fisheries of Korea, https://www.mof.go.kr/statPortal/). Various fecal sources, including sewage, farms, wildlife habitats, and harbors, may cause the fecal contamination of fisheries and shellfish farms, which are typically located near aquaculture areas in this region (6, 23, 40).

Microbial source tracking is an environmental monitoring method that has been applied globally to monitor the sources of feces in aquatic environments using a number of indicator microorganisms (10, 32, 39). In precise microbial source tracking studies, it is important to select microbial indicators that strongly correlate with fecal sources (8). Fecal indicator bacteria, such as total coliforms, fecal coliforms, and enterococci, are commonly used in water quality management worldwide (38). Although humans carry human-specific pathogens, animals are also important carriers or reservoirs of enteric human pathogens, such as *Salmonella* spp., enterohemorrhagic *Escherichia coli*, and *Vibrio* spp. (3, 28). Coliforms and enterococci may not be suitable for examining the origins of fecal sources due to their existence in human and animal feces (1, 18, 28). Therefore, host-specific microbial indicators are needed in order to accurately track fecal sources (2, 7).

*Bacteroides* spp. are obligate anaerobic bacteria, and many *Bacteroides* spp. have been detected in the feces of warm-blooded animals (35). Previous studies reported that several *Bacteroides* spp. had strong host or group specificities, and the 16S rRNA genes of host-specific *Bacteroides* spp. are promising markers for identifying human or animal fecal sources (17, 20). Moreover, *Bacteroides* spp. are suitable for estimating the time lapse of fecal inflow into aquatic environments due to their short period of viability after their release from hosts (43). Therefore, human-specific *Bacteroides* markers may be useful as molecular indicators of human-specific pathogens because they are highly abundant and have relatively high sensitivity and specificity to human fecal contamination (25, 27, 45). Human noroviruses, a major cause of non-bacterial acute gastroenteritis, only originate from human feces (5). Since their low sensitivity and high cost of detection limits their utility (37), human-specific *Bacteroides* markers may offer a useful alternative for monitoring and controlling norovirus contamination in aquaculture areas.

A large geographic information system (GIS) based on datasets, including population, land cover, and land use pattern data, may be used for precise and systematic fecal source tracking (26, 44). Land use patterns provide information on the spatial variability of a study area and the fecal sources in different types of land cover (26). Moreover, the patterns of land use and associated fecal sources significantly affect the surrounding aquatic environments (34). Therefore, the objectives of the present study were to (i) identify the distribution of host-specific *Bacteroides* markers in surface water and seawater samples from two aquaculture areas, (ii) predict potential fecal sources using a *Bacteroides* marker-combined geospatial map based on GIS, and (iii) investigate the relationship between human noroviruses and human-specific *Bacteroides* markers.

## Materials and Methods

### Sampling sites and water samples

**Fig. 1:**
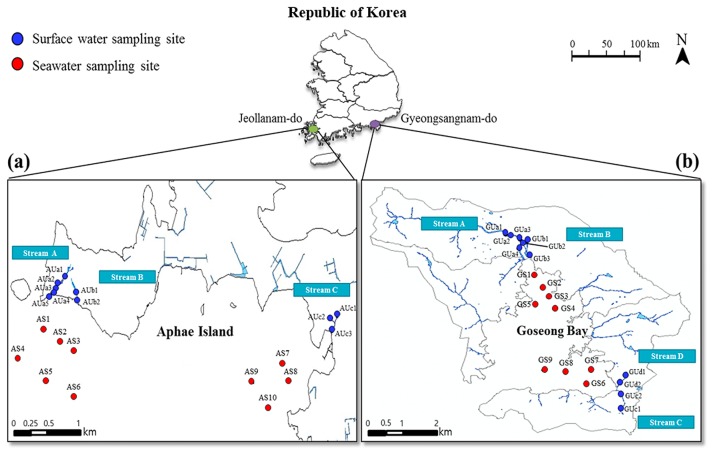
Sampling sites in this study: (a) Aphae Island. (b) Goseong Bay. Sampling site labels are based on the sampling site (A, Aphae Island; G, Goseong Bay), water type (U, surface water; S, seawater), and stream (A, B, C, or D), and numbers indicate the sampling location.

Two aquaculture areas (Aphae Island and Goseong Bay), located on the southern coast of Republic of Korea, exhibit distinct geographic characteristics. Aphae Island has a very rugged coastline and approximately 7,000 residents (Fig. 1). Several intensive livestock farms, which breed approximately 51,400 poultry, 8,900 pigs, and 2,900 ruminants, operate within the watershed of Aphae Island (Statistics Korea, http://kosis.kr). The daily capacity of sewage treatment facilities and the public sewage treatment connection rate (based on the percent of the population) are 70 m^3^ d^−1^ and 19.6%, respectively (National Sewer Information System, https://www.hasudoinfo.or.kr/). Goseong Bay is an enclosed geographic water feature into which seawater inflows relatively slowly due to the narrow entrance into the bay. Goseong Bay is a major area for oyster cultivation, accounting for approximately 13% of all oyster production in Republic of Korea, and has approximately 26,000 residents (11) (Ministry of Ocean and Fisheries of Korea, https://www.mof.go.kr/statPortal/). Intensive livestock farms, which breed approximately 105,000 poultry, 8,100 pigs, and 2,500 ruminants, are located within the watershed of Goseong Bay (Statistics Korea, http://kosis.kr). The daily capacity of sewage treatment facilities and the public sewage treatment connection rate are 13,000 m^3^ d^−1^ and 85.5%, respectively (National Sewer Information System, https://www.hasudoinfo.or.kr/).

In the present study, 240 water samples were collected six times, between March 2015 and January 2016, from various surface and seawater sampling sites located on Aphae Island and in Goseong Bay. Twenty sampling sites were selected on Aphae Island (10 surface water and 10 seawater sampling sites) and Goseong Bay (11 surface water and 9 seawater sampling sites) considering stream sites and sampling locations. During sampling, water temperature, pH, conductivity, salinity, and turbidity were measured using a YSI multi-parameter instrument (Yellow Springs Instruments, Yellow Springs, OH, USA). Furthermore, the various environmental parameters of each sampling site, including wind speed, precipitation on the sampling day (Prep-0), and total precipitation for the 3 d preceding the sampling day (Prep-3), were obtained from the Korea Meteorological Administration (http://www.kma.go.kr).

### Sample processing and nucleic acid extraction

Water samples were collected in sterilized bottles and filtered using polyvinylidene difluoride membranes (pore size of 0.22 μm, diameter of 47 mm, Millipore, Cork, Ireland). Filters were kept at −80°C until nucleic acid extraction using a PowerWater DNA isolation kit (MO BIO Laboratories, Carlsbad, CA, USA) following the manufacturer’s instructions. The final eluents (100 μL each) were stored at −20°C until used.

In order to detect human noroviruses, 100 L of each water sample was filtered using NanoCeram cartridge filters (Argonide Corp., Sanford, FL, USA) as described previously with minor modifications (9). In order to elute noroviruses from the filters, 0.5 L of elution buffer containing 1.5% beef extract (BD Biosciences, San Jose, CA, USA), 0.05 M glycine (Duchefa, St. Louis, MO, USA), and 1 M NaOH (pH 9.5) was used. After a 5-min incubation, the pH of the eluent was adjusted to 3.5 using 1 M HCl, and the precipitate was acquired via centrifugation at 2,500×*g* at 4°C for 15 min. The precipitate was completely resuspended in 0.15 M sodium phosphate (pH 9.0–9.5), and centrifuged at 10,000×*g* at 4°C for 10 min. After adjusting to a pH of 7.0–7.5 using 1 M HCl, the supernatant was filtered using a syringe filter (pore size of 0.22 μm, Millipore) and stored at −80°C. Viral nucleic acid extraction was performed using the QIAamp Viral RNA Mini Kit (Qiagen, Hilden, Germany) according to the manufacturer’s instructions. The final eluents (60 μL each) were stored at −20°C until used.

### Quantification of *Bacteroides* markers

Human-specific *Bacteroides dorei* strain KCTC 5446 (Korean Collection for Type Cultures, Jeongeup, Korea) was cultivated in tryptic soy agar (BD Biosciences) with hemin (Sigma-Aldrich, St Louis, MO, USA) and menadione (Sigma-Aldrich) under anaerobic conditions at 37°C. The bacteria were harvested via centrifugation at 14,000×*g* at 4°C for 5 min. The bacterial genome was extracted using the G-spin Genomic DNA Extraction Kit for Bacteria (Intron Biotechnology, Seongnam, Korea) following the manufacturer’s instructions, and stored at −20°C until used. The genomes of animal-specific *Bacteroides* were extracted from 200 mg of chicken, pig, and cow fecal samples. The bacterial genomes from the fecal sample of each animal were extracted with the QIAamp DNA Stool Mini Kit (Qiagen) with minor modifications. Briefly, InhibitEX buffer-treated (1 mL) fecal samples were heated at 95°C for 5 min, and then bead-beaten with 0.1-mm beads for 10 min as described previously (48). The bacterial genomes were subjected to a polymerase chain reaction (PCR) using the ABI PRISM GeneAmp PCR system 9700 (Applied Biosystems, Forster City, CA, USA). Table 1 summarizes the primers and probes for the specific *Bacteroides* markers used in the present study. The reaction mixture consisted of 2.5 μL of template DNA, 2.5 μL of 10× PCR buffer with 25 mM MgCl_2_, 1 μL of 10 mM dNTP mix, and 0.25 μL of Taq polymerase (Cosmo Genetech Corp., Seoul, Korea). PCR was performed under the following conditions: initial denaturation at 95°C for 3 min, 30 cycles of denaturation at 95°C for 15 s, annealing at 60°C for 15 s, extension at 72°C for 45 s, and final extension at 72°C for 3 min. PCR products were purified using the QIAquick PCR Purification Kit (Qiagen), and ligated into the pGEM-T Easy Vector System (Promega Corp., Madison, WI, USA) with *E. coli* DH5*α* cells following the manufacturer’s instructions. Plasmid DNA was extracted from the transformed cells using a LaboPass Plasmid DNA Purification Kit (Cosmo Genetech Corp.). Plasmid DNA concentrations were measured using the NanoDrop spectrophotometer ND-1000 (NanoDrop Technologies, Wilmington, DE, USA), and the total copies of the markers were computed to quantify the *Bacteroides* markers, as described previously (22).

In order to assess the *Bacteroides* marker concentrations, real-time quantitative PCR was performed using an Applied Biosystems 7300 Real-time PCR system (Applied Biosystems). The reaction mixture consisted of 2 μL of template DNA, 10 μL of 2× TaqMan Universal PCR Master Mix (Applied Biosystems), and appropriate final concentrations of each primer and probe (Table 1). Real-time PCR was performed in duplicate under the following conditions: 1 cycle at 95°C for 10 min, 45 cycles of 95°C for 15 s, and 60°C for 1 min. All water samples were diluted 10-fold to mitigate PCR inhibitors. As an internal quality control, negative controls (*i.e.*, no template DNA) were performed for each experiment.

### Detection of noroviruses using real-time quantitative RT-PCR

Duplex real-time reverse transcriptase PCR was performed to estimate the concentrations of norovirus genogroup I (GI) and genogroup II (GII) in surface and seawater samples (16) (Ministry of Food and Drug Safety. 2013. Guideline for investigation on the cause of food poisoning, Chapter 5. Chungcheonbuk-do, Republic of Korea, in Korean). Quantitative reverse transcription (RT-q) PCR assays were executed with a C1000 Thermal Cycler CFX96 Real-time PCR System (Bio-Rad, Hercules, CA, USA). The RT-qPCR reaction mixture consisted of 5 μL of template RNA, 400 nM of each primer (Table 1), 200 nM of each probe (Table 1), 12.5 μL of 2× RT-PCR buffer, 0.5 μL of 25× RT-PCR enzyme mix, and 1.5 μL of the detection enhancer using the AgPath-ID One-Step RT-PCR Kit (Thermo Fisher Scientific, Waltham, MA, USA). The reaction mixtures were incubated at 45°C for 30 min for the reverse transcription reaction. RT-qPCR was performed under the following conditions: initial denaturation at 95°C for 10 min, 45 cycles of 95°C for 10 s, and 56°C for 1 min. Ten-fold-diluted norovirus RNA-positive controls (AccuPower Norovirus Real-time RT-PCR Kit, Bioneer, Daejeon, Korea) were used to quantify the viral copy number.

### Detection of total and fecal coliforms

Total and fecal coliforms in surface and seawater samples were assessed using the standard most probable number (MPN) method (31). Five tubes of lauryl tryptose broth (BD Biosciences) were inoculated with diluted water samples and incubated at 35°C for 48 h. In order to distinguish between total and fecal coliforms, gas-producing samples were re-inoculated in brilliant green bile broth (Oxoid, Hampshire, UK) at 35°C for 48 h and in *E. coli* broth (BD Biosciences) at 44.5°C for 24 h. Coliform concentrations were calculated based on the MPN table.

### Host-specific *Bacteroides* marker-combined geospatial analysis

The host-specific *Bacteroides* marker-combined geospatial analysis was performed using ArcGIS ver. 10.2.2 (ESRI, Redlands, CA, USA). Thematic maps denoting administrative divisions, land-use patterns, and watersheds near the sampling sites were obtained from the Korea Water Resources Management Information System (http://www.wamis.go.kr).

### Statistical analysis

IBM SPSS ver. 18.0.0 (SPSS, Chicago, IL, USA) and GraphPad Prism software ver. 5.0 (GraphPad Software, San Diego, CA, USA) were used for data analyses. The environmental data obtained in the present study were not normally distributed (Shapiro-Wilk test); therefore, the nonparametric test was used to compare the values of environmental parameters between the two sampling areas. Prior to the statistical analyses, the concentrations of *Bacteroides* markers, noroviruses, and coliforms were log-transformed (log_10_ [copies+1]), as described previously (13). Data are expressed as means±standard deviation (SD) and analyzed using the Mann-Whitney *U* test. *P* values <0.05 were considered to indicate significance. In correlation analyses between noroviruses and microbial indicators, Spearman’s correlation coefficient (*r*) was calculated.

## Results

### Environmental parameters in water samples

Table 2 shows the results of the environmental parameters in water samples from two sampling areas. The median values of water temperature for both surface and seawater from Goseong Bay, 19.4°C and 16.1°C, respectively, were higher than those from Aphae Island. Surface water from Goseong Bay showed the lowest median values of pH (7.3), conductivity (116 μS cm^−1^), and salinity (0.16 practical salinity unit). Surface water from Goseong Bay showed the highest median value of turbidity (10.80 nephelometric turbidity unit). The highest median values of wind speed (3.5 m s^−1^) and Prep-3 (8.4 mm) were recorded during sampling for surface water from Aphae Island. The median values for most environmental parameters significantly differed between the two sampling areas (*P*<0.05). Only wind speed and Prep-3, and salinity and Prep-0 were similar in surface water samples and seawater samples from both sampling areas, respectively.

### Concentrations and occurrences of host-specific *Bacteroides* markers in water samples

Table 3 summarizes the concentrations and occurrences of host-specific *Bacteroides* markers in water samples from two sampling areas. Surface and seawater samples from Aphae Island both showed significantly higher mean concentrations of the human-specific *Bacteroides* marker than those from Goseong Bay (*P*<0.05). Surface water samples from Aphae Island showed the highest mean concentration (1.1 log_10_ copies L^−1^) and occurrence (25%) of the human-specific *Bacteroides* marker. Moreover, the poultry-specific *Bacteroides* marker was detected at more than 35% in all types of water samples, and surface water samples from Aphae Island showed the highest mean concentration (1.2 log_10_ copies L^−1^) and occurrence (50%). The mean concentration of the pig-specific *Bacteroides* marker was similar in all water samples, while the ruminant-specific *Bacteroides* marker was rarely detected.

### Seasonal and spatial variations in host-specific *Bacteroides* markers

**Fig. 2:**
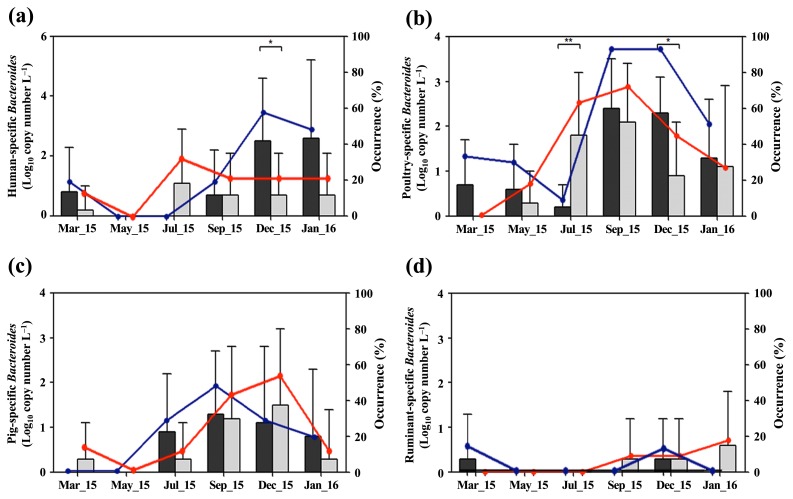
Mean concentrations (black bar, Aphae Island; gray bar, Goseong Bay) and occurrences of host-specific *Bacteroides* markers (blue circle, Aphae Island; red circle, Goseong Bay) in surface water samples obtained over the sampling period: (a) human-specific *Bacteroides* marker, (b) poultry-specific *Bacteroides* marker, (c) pig-specific *Bacteroides* marker, and (d) ruminant-specific *Bacteroides* marker. Data are expressed as the means±standard deviation (SD) and asterisks indicate significant differences (*P<0.05, **P<0.01; Mann-Whitney *U* test).

Fig. 2 shows the distribution of host-specific *Bacteroides* markers in surface water samples over the sampling period. Aphae Island samples showed higher concentrations of the human-specific *Bacteroides* marker than Goseong Bay samples in March 2015, and between September 2015 and January 2016 (Fig. 2a). Aphae Island samples showed the highest mean concentration (2.4 log_10_ copies L^−1^) and occurrence (60%) (6 of 10) of the human-specific *Bacteroides* marker in December 2015, which was significantly higher than that in Goseong Bay samples (*P*<0.05). Moreover, Aphae Island samples showed a higher mean concentration of the poultry-specific *Bacteroides* marker than Goseong Bay samples over the entire sampling period, with the exception of samples in July 2015 (Fig. 2b). More than 1.0 log_10_ copies L^−1^ of the pig-specific *Bacteroides* marker were detected in all water samples in both sampling areas between September and December 2015 (Fig. 2c). The ruminant-specific *Bacteroides* marker was rarely detected during the sampling period (Fig. 2d).

**Fig. 3:**
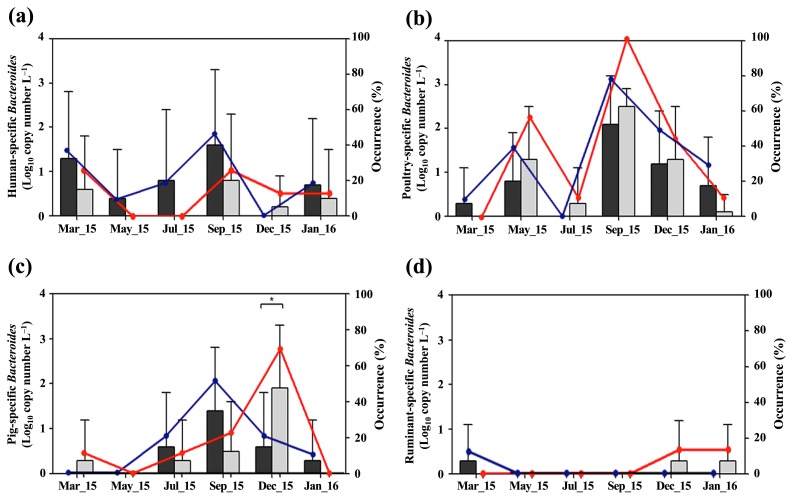
Mean concentrations (black bar, Aphae Island; gray bar, Goseong Bay) and occurrences of host-specific *Bacteroides* markers (blue circle, Aphae Island; red circle, Goseong Bay) in seawater samples obtained over the sampling period: (a) human-specific *Bacteroides* marker, (b) poultryspecific *Bacteroides* marker, (c) pig-specific *Bacteroides* marker, and (d) ruminant-specific *Bacteroides* marker. Data are expressed as the means±SD and asterisks indicate significant differences (*P<0.05; Mann-Whitney *U* test).

Fig. 3 shows the distribution of host-specific *Bacteroides* markers in seawater samples during the sampling period. Aphae Island samples showed higher concentrations of the human-specific *Bacteroides* marker than Goseong Bay samples over the entire sampling period, with the exception of samples obtained in December 2015 (Fig. 3a). In September 2015, Aphae Island samples showed the highest mean concentration (1.6 log_10_ copies L^−1^) and occurrence (60%) (6 of 10) of the human-specific *Bacteroides* marker. Meanwhile, Goseong Bay samples showed higher concentrations of the poultry-specific *Bacteroides* marker than Aphae Island samples between May and December 2015 (Fig. 3b). In September 2015, Goseong Bay samples showed the highest mean concentration (2.5 log_10_ copies L^−1^) and occurrence (100%) (9 of 9) of the poultry-specific *Bacteroides* marker. Goseong Bay samples showed the highest mean concentration (1.9 log_10_ copies L^−1^) and occurrence (67%) (7 of 9) of the pig-specific *Bacteroides* marker, which was significantly higher than that of Aphae Island samples (*P*<0.05) (Fig. 3c). Only a few seawater samples were positive for the ruminant-specific *Bacteroides* marker (Fig. 3d).

### Host-specific *Bacteroides* marker-combined geospatial analysis

**Fig. 4:**
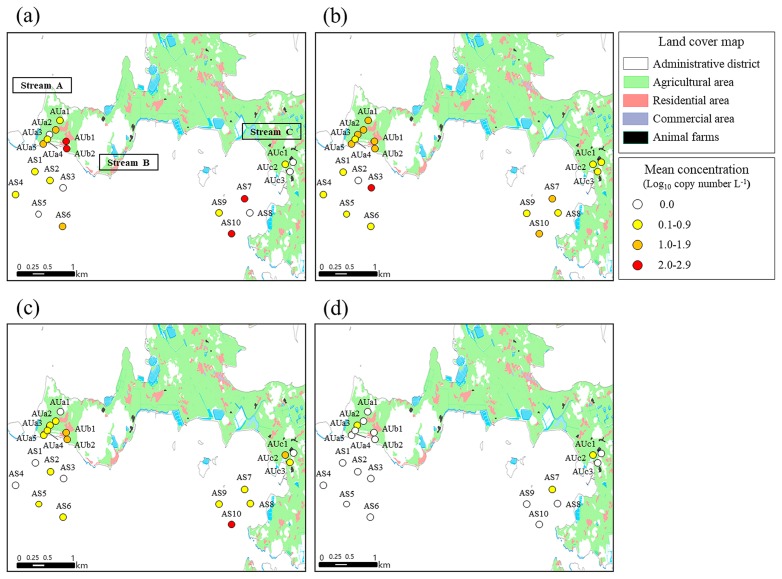
Host-specific *Bacteroides* marker-combined geospatial map including land-use patterns on Aphae Island: (a) human-specific *Bacteroides* marker, (b) poultry-specific *Bacteroides* marker; (c) pig-specific *Bacteroides* marker, and (d) ruminant-specific *Bacteroides* marker.

Fig. 4 summarizes the results of the host-specific *Bacteroides* marker-combined geospatial analysis considering land-use patterns on Aphae Island. The surface sampling sites in Stream B, located near a residential area, showed a higher mean concentration of the human-specific *Bacteroides* marker (Fig. 4a). Sites AUb1 and AUb2 showed 2.7 log_10_ copies L^−1^ and 2.8 log_10_ copies L^−1^ of the human-specific *Bacteroides* marker, respectively. Sites AS7 and AS10 showed more than 2.0 log_10_ copies L^−1^ of the human-specific *Bacteroides* marker, as well as the highest occurrence (67%) (4 out of 6). The surface water sampling sites in Streams A and B showed more than 1.0 log_10_ copies L^−1^ of the poultry-specific *Bacteroides* marker, with the exception of Site AUa3 (Fig. 4b). Site AS3, located near Stream B, showed the highest poultry-specific *Bacteroides* marker concentration (2.1 log_10_ copies L^−1^) with an occurrence of 83% (5 out of 6). Moreover, Site AUc2, located near a pig barn, showed 1.9 log_10_ copies L^−1^ of the pig-specific *Bacteroides* marker with an occurrence of 50% (3 of 6), while Site AS10 showed the highest pig-specific *Bacteroides* marker concentration (2.0 log_10_ copies L^−1^) with an occurrence of 67% (4 of 6) (Fig. 4c).

**Fig. 5:**
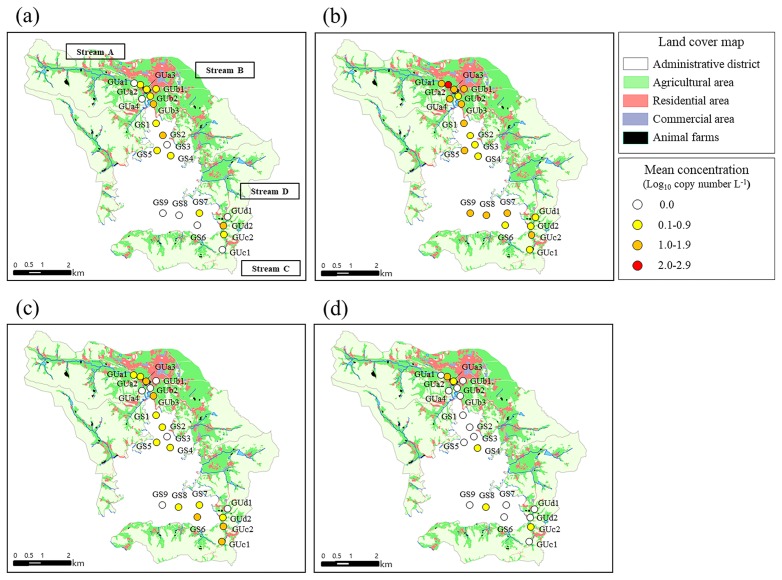
Host-specific *Bacteroides* marker-combined geospatial map including land-use patterns in Goseong Bay: (a) human-specific *Bacteroides* marker, (b) poultry-specific *Bacteroides* marker, (c) pig-specific *Bacteroides* marker, and (d) ruminant-specific *Bacteroides* marker.

Fig. 5 shows the results of the host-specific *Bacteroides* marker-combined geospatial analysis in Goseong Bay. Site GUb3, located downstream of a residential area, showed the highest mean concentration of the human-specific *Bacteroides* marker (1.7 log_10_ copies L^−1^) with an occurrence of 50% (3 of 6) (Fig. 5a). The surface water sampling sites in Streams A and B showed more than 1.0 log_10_ copies L^−1^ of the poultry-specific *Bacteroides* marker, with the exception of Site GUb3 (Fig. 5b). Sites GS1 and GS5, located near Streams A and B, showed more than 1.0 log_10_ copies L^−1^ of the poultry-specific *Bacteroides* marker and an occurrence of 50% (3 of 6). Moreover, three seawater sampling sites (GS7, GS8, and GS9) located near streams C and D showed more than 1.0 log_10_ copies L^−1^ of the poultry-specific *Bacteroides* marker. In addition, Site GUa3 showed the highest mean concentration of the pig-specific *Bacteroides* marker (1.6 log_10_ copies L^−1^) with an occurrence of 50% (3 of 6) (Fig. 5c). The water sampling sites in stream C showed more than 1.0 log_10_ copies L^−1^ of the pig-specific *Bacteroides* marker. Site GUa2, located in Stream A, showed the highest concentration of the ruminant-specific *Bacteroides* marker (1.1 log_10_ copies L^−1^) with an occurrence of 33% (2 of 6) (Fig. 5d).

Table S1 summarizes the concentrations of host-specific *Bacteroides* markers in the streams located at Aphae Island and Goseong Bay over the sampling period. Between December 2015 and January 2016, Stream A samples from Aphae Island showed more than 5.0 log_10_ copies L^−1^ of the human-specific *Bacteroides* marker. Four Stream B samples from Aphae Island showed more than 3.7 log_10_ copies L^−1^ of the human-specific *Bacteroides* marker. With the exception of March 2015, the poultry-specific *Bacteroides* marker was detected in Stream A samples from Aphae Island. Moreover, Stream B and A samples from Goseong Bay showed the highest occurrences (5 of 6) (83%) of the human-specific *Bacteroides* marker and poultry-specific *Bacteroides* marker, respectively.

### Concentrations of noroviruses and coliforms in water samples

Table 4 summarizes the concentrations and occurrences of noroviruses and coliforms in surface water samples. Aphae Island samples showed significantly higher mean concentrations of noroviruses than those of Goseong Bay samples (*P*<0.001), which were 1.4 log_10_ copies L^−1^ of norovirus GI with an occurrence of 37% (22 of 60) and 1.9 log_10_ copies L^−1^ of norovirus GII with an occurrence of 43% (26 of 60). In contrast, the mean coliform concentration was higher in Goseong Bay than in Aphae Island (*P*<0.05). Noroviruses and coliforms were not detected in any of the seawater samples.

### Correlations among host-specific *Bacteroides* markers, noroviruses, and coliforms in water samples

Table 5 shows the results of the correlation analysis of host-specific *Bacteroides*, noroviruses, and coliforms in surface water samples. The concentration of noroviruses (GI and GII) correlated more strongly with the concentration of the human-specific *Bacteroides* marker (*r*=0.337; *P*<0.001) than total (*r*=0.304; *P*<0.01) or fecal coliforms (*r*=0.223; *P*<0.01). In contrast, noroviruses (GI and GII) showed a negative correlation with the pig-specific *Bacteroides* marker (*r*=−0.173; *P*<0.05), and exhibit no correlations with poultry- or ruminant-specific *Bacteroides* markers. Neither coliform correlated with the human-specific *Bacteroides* marker.

## Discussion

In the present study, marked differences were observed in several environmental parameters between the two sampling areas (Table 2). A previous study suggested that environmental parameters indicating the water condition, such as temperature, conductivity, pH, and salinity, significantly affected the concentrations of qPCR markers for fecal indicator bacteria (29). Moreover, as previously reported, rainfall events may be a major transporter of feces in water environments (24). Therefore, several environmental factors closely related to rainfall events, such as Prep-0 and Prep-3, may also affect the concentrations of host-specific *Bacteroides* in both areas. However, in future studies, water quality data, such as ammonium, dissolved oxygen, and chemical oxygen demands, also need to be investigated due to their potential effects on the concentrations of host-specific *Bacteroides*.

All of the water samples examined in the present study showed a more than 10% of occurrence of the human-, poultry-, and pig-specific *Bacteroides* markers, indicating that *Bacteroides* spp. markers were widely present in the studied areas as well as their suitability as markers. The mean concentration and occurrence of the poultry- and pig-specific *Bacteroides* markers in surface water samples from Aphae Island were higher than those from Goseong Bay (Table 3), which may have been affected by the number of animals nearby (Statistics Korea, http://kosis.kr/). However, human-specific *Bacteroides* marker concentrations were significantly higher in Aphae Island samples than in Goseong Bay samples (*P*<0.05), even though Goseong Bay had a greater residential population than Aphae Island. These results may have been due to the public sewage treatment connect rate, which is higher in Goseong Bay (85.5%) than in Aphae Island (19.6%) (National Sewer Information System, https://www.hasudoinfo.or.kr/). Residents unconnected to the public sewage treatment system may directly or indirectly affect the aquatic environment (Gyeongnam development institute. 2011. A study on improvement of water quality in Milyanggang. Gyeongsangnam-do, Republic of Korea, in Korean). Therefore, improvements in the public sewage treatment system connection rate need to be considered in order to prevent the fecal contamination of aquatic environments (14).

In the present study, high mean concentrations of host-specific *Bacteroides* markers were often detected between autumn and early winter (September to December 2015) regardless of the geographical condition or water type (Fig. 2 and 3). Temperature is a major factor affecting the persistence of *Bacteroides* spp. in the environment (4). Moreover, *Bacteroides*-originating genetic markers exhibit greater persistence at low temperatures in surface and seawater because low temperatures prevent *Bacteroides* 16S rRNA gene decay (33, 46). Therefore, temperature changes in water need to be considered in microbial source tracking using host-specific *Bacteroides* markers because changes in temperature may result in the over- or underestimation of fecal contaminants (19). However, due to the number of fecal sources, further longitudinal research with mass balance and load analyses of fecal contamination is necessary in order to track host-specific *Bacteroides* markers in the study areas.

The host-specific *Bacteroides* marker-combined geospatial analysis revealed the up-to-downstream gradient of fecal contamination, indicating that surface water affects the fecal contamination of seawater (Fig. 4 and 5). Table S1 also showed the temporal tendencies of the high concentrations of host-specific *Bacteroides* markers in autumn to winter (September 2015 to January 2016) in stream samples. However, streams located near residential areas, including Stream B in Aphae Island and Streams A and B in Goseong Bay, generally showed higher concentrations of human, poultry, and pig-specific *Bacteroides* markers over the sampling period than other streams. Therefore, the land use pattern may be closely related to the concentrations of host-specific *Bacteroides* markers. According to the land use pattern, the concentrations of host-specific *Bacteroides* markers showed clear variations in both study areas. Previous studies reported that the concentrations of fecal indicators were high in water bodies near residential areas (41, 47). In the present study, the sampling sites located near densely populated residential areas showed higher mean concentrations of the human-specific *Bacteroides* marker. The sampling sites near residential areas also showed high mean concentrations of poultry- and pig-specific *Bacteroides* markers (Fig. 4 and 5). This may be explained by the characteristics of these areas, at which small-scale livestock breeding occurs in residential areas, and high concentrations of *Bacteroides* markers may have been observed due to the application of fertilizers containing human or animal feces (30, 42). Furthermore, the results obtained for the high host-specific *Bacteroides* marker concentrations in populated areas indicated that transportation is important for the fecal contamination of study areas. Therefore, further studies using *Bacteroides* marker-combined geospatial analyses need to be designed with the major routes and daily loads of transportation for precise fecal source tracking in aquaculture areas. The results of the present study demonstrated that the host-specific *Bacteroides* marker-combined geospatial analysis has potential as a useful tool for predicting the environmental effects of potential fecal sources.

Moreover, our results demonstrated that the human-specific *Bacteroides* marker correlated more strongly with human noroviruses than total or fecal coliforms (*P*<0.001) (Table 5). Previous studies reported that the presence of several enteric viruses, including human adenoviruses, polyomaviruses, and noroviruses, correlated with human-associated *Bacteroides* spp. in aquatic environments (15, 27). Therefore, since human-specific *Bacteroides* and human noroviruses both share strong human specificity (25), human-specific *Bacteroides* markers may be used to trace the waterborne transmission of human noroviruses originating from virus-contaminated feces.

The results of the present study indicate that the host-specific *Bacteroides* markers used herein are suitable genetic markers for fecal source tracking regardless of geographical differences, such as those between open islands (Aphae Island) and enclosed bays (Goseong Bay). Despite seasonal trends in concentrations and occurrences, the host-specific *Bacteroides* marker-combined geospatial analysis showed a clear transition in fecal contamination. Furthermore, the human-specific *Bacteroides* marker correlated with human noroviruses. Therefore, host-specific *Bacteroides* markers with an advanced geospatial analysis appear to be useful for tracking fecal contamination in aquatic environments.

## Supplementary Material



## Figures and Tables

**Table 1:** Primers and probes used for the real-time PCR in this study

Target	Primer and probe	Sequence (5′→3′)	Final concentration (nM)	Reference(s)
Human-specific *Bacteroides*	HF183_F	ATCATGAGTTCACATGTCCG	1000	12
	HF183_R	TACCCCGCCTACTATCTAATG	1000	
	HF183_P	(FAM) CTGAGAGGAAGGTCCCCCACATTGGA (TAMRA)	80	
Poultry-specific *Bacteroides*	qCD362_F	AATATTGGTCAATGGGCGAGAG	200	19
	qCD464_R	CACGTAGTGTCCGTTATTCCCTTA	200	
	qCD394_P	(FAM) TCCTTCACGCTACTTGG (NFQ-MGB)	200	
Pig-specific *Bacteroides*	Pig-2-Bac_F	GCATGAATTTAGCTTGCTAAATTTGAT	300	28
	Pig-2-Bac_R	ACCTCATACGGTATTAATCCGC	300	
	Pig-2-Bac_P	(VIC) TCCACGGGATAGCC (NFQ-MGB)	200	
Ruminant-specific *Bacteroides*	BacR_F	GCGTATCCAACCTTCCCG	100	35
	BacR_R	CATCCCCATCCGTTACCG	500	
	BacR_P	(FAM) CTTCCGAAAGGGAGATT (NFQ-MGB)	100	
Norovirus GI	COG1F	CGYTGGATGCGNTTYCATGA	400	16, Ministry of Food and Drug Safety. 2013. Guideline for investigation on the cause of food poisoning, Chapter 5. Chungcheonbuk-do, Republic of Korea, in Korean
	COG1R	CTTAGACGCCATCATCATTYAC	400	
	RING1(a)-TP	(FAM) AGATYGCGATCYCCTGTCCA (TAMRA)	200	
Norovirus GII	BPO-13	AICCIATGTTYAGITGGATGAG	400	Ministry of Food and Drug Safety. 2013. Guideline for investigation on the cause of food poisoning, Chapter 5. Chungcheonbuk-do, Republic of Korea, in Korean
	BPO-13N	AGTCAATGTTTAGGTGGATGAG	400	
	BPO-14	TCGACGCCATCTTCATTCACA	400	
	BPO-18	(VIC) CACRTGGGAGGGCGATCGCAATC (TAMRA)	200	

**Table 2:** Summary of environmental parameters

Parameter	Surface water		Seawater	
	
	Aphae Island	Goseong Bay	Aphae Island	Goseong Bay
Water temperature (°C)	14.7 8.2 (17.0)^a^	17.9±6.0 (19.4)	14.4±6.4 (13.2)	15.7±7.7 (16.1)
pH	7.8±0.6 (7.9)	7.3±0.6 (7.3)	7.8±0.4 (7.9)	7.8±0.6 (8.1)
Conductivity (μS cm^−1^)	3,150±8,750 (760)	961±3,120 (116)	49,500±1,440 (49,300)	36,400±7,710 (36,800)
Salinity (psu^b^)	1.88±5.59 (0.37)	1.49±4.60 (0.16)	32.26±1.08 (32.13)	32.42±1.53 (33.01)
Turbidity (NTU^c^)	15.01±15.85 (10.80)	7.04±5.24 (5.86)	8.91±6.31 (7.80)	1.99±1.02 (1.87)
Wind speed (m s^−1^)	3.5±1.4 (3.5)	3.2±1.1 (2.7)	2.9±1.7 (2.5)	2.0±0.3 (1.9)
Prep-0^d^ (mm)	0.8±1.7 (0.0)	12.9±27.5 (0.0)	7.8±17.0 (0.0)	0.6±1.1 (0.0)
Prep-3^e^ (mm)	25.1±39.8 (8.4)	12.8±18.0 (4.3)	9.1±9.0 (6.6)	1.1±1.3 (0.5)

a Mean±standard deviation (median)

b Practical salinity unit

c Nephelometric turbidity unit

d Cumulative precipitation on the sampling day

e Total cumulative precipitation from 3 d before the sampling day

**Table 3:** Concentrations and occurrences of host-specific *Bacteroides* markers in surface and seawater samples from Aphae Island and Goseong Bay

Host-specific *Bacteroides* markers	Aphae Island				Goseong Bay			
	
	Surface water (*n*=60)		Seawater (*n*=60)		Surface water (*n*=66)		Seawater (*n*=54)	
			
	Mean^a^±SD^b^ (range)	Occurrence (%)	Mean±SD (range)	Occurrence (%)	Mean±SD (range)	Occurrence (%)	Mean±SD (range)	Occurrence (%)
Human-specific	1.1±1.9 (ND^c^-5.7)	15 (25)	0.8±1.5 (ND-4.0)	14 (23)	0.6±1.3 (ND-4.2)	10 (15)	0.3±1.0 (ND-3.7)	6 (11)
Poultry-specific	1.2±1.3 (ND-3.2)	30 (50)	0.9±1.2 (ND-3.4)	21 (35)	1.0±1.4 (ND-4.2)	25 (38)	0.9±1.2 (ND-3.1)	21 (39)
Pig-specific	0.7±1.3 (ND-4.6)	13 (22)	0.5±1.1 (ND-3.1)	10 (17)	0.6±1.3 (ND-3.7)	12 (18)	0.5±1.1 (ND-3.2)	10 (19)
Ruminant-specific	0.1±0.6 (ND-3.2)	2 (3)	ND^c^	0 (0)	0.2±0.8 (ND-3.5)	4 (6)	0.1±0.5 (ND-2.9)	2 (4)

a Unit: log_10_ copy number L^−1^

b SD: Standard deviation

c ND: Not detected

**Table 4:** Concentrations and occurrences of noroviruses and coliforms in surface water samples from Aphae Island and Goseong Bay

Types	Aphae island		Goseong bay	
	
	Surface water (*n*=60)		Surface water (*n*=66)	
	
	Mean±SD (range)^a^	Occurrence (%)	Mean±SD (range)	Occurrence (%)
Norovirus GI	1.4±1.9 (ND-5.6)	22 (37)	0.8±1.7 (ND-5.9)	13 (20)
Norovirus GII	1.9±2.2 (ND-5.5)	26 (43)	0.8±1.6 (ND-5.7)	13 (20)
Total coliforms	4.3±1.2 (1.7–6.2)	60 (100)	4.4±1.1 (1.3–8.2)	66 (100)
Fecal coliforms	3.2±1.4 (1.3–6.2)	60 (100)	3.8±1.3 (1.3–8.2)	66 (100)

a Unit: log_10_ copy number L^−1^ (noroviruses); log_10_ MPN L^−1^ (coliforms)

**Table 5:** Spearman’s correlation coefficients among *Bacteroides* markers, noroviruses, and coliforms in surface water samples from Aphae Island and Goseong Bay

Types	Human-specific *Bacteroides* marker	Poultry-specific *Bacteroides* marker	Pig-specific *Bacteroides* marker	Ruminant-specific *Bacteroides* marker	Norovirus GI	Norovirus GII	Norovirus GI+GII	Total coliforms	Fecal coliforms
Human-specific *Bacteroides* marker	1.000								
Poultryspecific *Bacteroides* marker	0.259**^a^	1.000							
Pig-specific *Bacteroides* marker	0.184*	0.132	1.000						
Ruminant-specific *Bacteroides* marker	0.125	0.069	0.192*	1.000					
Norovirus GI	0.263**	−0.059	−0.135	−0.046	1.000				
Norovirus GII	0.374***	0.010	−0.196*	−0.061	0.877***	1.000			
Norovirus GI+GII	0.337***	−0.019	−0.173*	−0.067	0.934***	0.969***	1.000		
Total coliforms	0.078	−0.112	−0.028	−0.035	0.299**	0.296**	0.304**	1.000	
Fecal coliforms	0.124	−0.128	0.050	−0.032	0.0236**	0.214*	0.233**	0.835***	1.000

a Asterisks indicate significant differences (*P<0.05, **P<0.01, ***P<0.001)
