# Supplementary material for: Host-Specific Bacteroides Markers-Based Microbial Source Tracking in Aquaculture Areas
Source: Microbes Environ. 2018 Jun 1;33(2):151–61. doi: 10.1264/jsme2.ME17166 (PMC6031393; doi:10.1264/jsme2.ME17166)
Supplement: Supplementary file 1 [file 33_151_s1.pdf]

Table S1. The concentrations of host-specific *Bacteroides* markers in the streams from Aphae Island and Goseong Bay over the sampling period

Host-specific *Bacteroides* markers	Sampling period (Month/year)	Aphae Island			Goseong Bay			
		Stream A	Stream B	Stream C	Stream A	Stream B	Stream C	Stream D
Human-specific	Mar/2015	ND[a]	3.8 ± 2.7[b]	ND	ND	2.2 ± 2.4	ND	ND
	May/2015	ND	ND	ND	ND	ND	ND	ND
	Jul/2015	ND	ND	ND	3.4 ± 3.7	3.6 ± 3.8		3.6 ± 3.8
	Sep/2015	ND	3.7 ± 2.6	ND	ND	3.2 ± 3.4	3.4 ± 3.6	ND
	Dec/2015	5.0 ± 5.4	4.0 ± 3.8	3.1 ± 3.3	ND	2.8 ± 3.1	ND	3.8 ± 4.0
	Jan/2016	5.2 ± 5.3	5.0 ± 4.3	ND	2.5 ± 2.8	3.7 ± 4.0	ND	ND
Poultry-specific	Mar/2015	ND	2.3 ± 1.8	1.7 ± 2.0	ND	ND	ND	ND
	May/2015	2.1 ± 2.3	0.8 ± 1.0	ND	1.7 ± 2.0	0.1 ± 0.3	ND	ND
	Jul/2015	0.8 ± 1.2	ND	ND	3.0 ± 2.9	2.5 ± 2.5	2.4 ± 2.5	2.7 ± 2.8
	Sep/2015	2.8 ± 2.8	2.7 ± 2.6	3.0 ± 2.4	3.1 ± 3.3	2.7 ± 2.7	2.8 ± 2.9	2.8 ± 2.8
	Dec/2015	2.8 ± 2.8	2.5 ± 2.5	2.1± 2.2	2.6 ± 2.7	2.1 ± 2.3	0.4 ± 0.6	1.0 ± 1.2
	Jan/2016	2.7 ± 2.9	2.5 ± 2.6	ND	3.9 ± 3.9	ND	ND	ND
Pig-specific	Mar/2015	ND	ND	ND	2.2 ± 2.5	ND	ND	ND
	May/2015	ND	ND	ND	ND	ND	ND	ND
	Jul/2015	2.3 ± 2.6	3.0 ± 3.1	1.8 ± 2.1	ND	ND	2.5 ± 2.7	ND
	Sep/2015	2.5 ± 2.6	2.9 ± 2.9	ND	2.8 ± 2.9	3.2 ± 3.5	3.0 ± 3.2	ND
	Dec/2015	ND	2.7 ± 2.9	4.2 ± 4.4	2.8 ± 3.1	2.6 ± 2.8	3.4 ± 2.6	3.1 ± 3.3
	Jan/2016	ND	2.8 ± 3.0	3.9 ± 4.2	3.1 ± 3.4	ND	ND	ND
Ruminant-specific	Mar/2015	2.5 ± 2.8	ND	ND	ND	ND	ND	ND
	May/2015	ND	ND	ND	ND	ND	ND	ND
	Jul/2015	ND	ND	ND	ND	ND	ND	ND
	Sep/2015	ND	ND	ND	ND	ND	2.8 ± 2.9	ND
	Dec/2015	ND	ND	2.5 ± 2.7	2.6 ± 2.9	ND	ND	ND
	Jan/2016	ND	ND	ND	3.0 ± 3.2	ND	ND	ND

[a] ND: Not detected

[b] Median ± standard deviation; Unit: log_{10} copies/L
